# Transforming Growth Factor β and Insulin Signal Changes in Stromal Fibroblasts of Individual Keratoconus Patients

**DOI:** 10.1371/journal.pone.0106556

**Published:** 2014-09-23

**Authors:** James Foster, Wai-Hong Wu, Sherri-Gae Scott, Mehak Bassi, Divya Mohan, Yassine Daoud, Walter J. Stark, Albert S. Jun, Shukti Chakravarti

**Affiliations:** 1 Department of Medicine, Johns Hopkins University School of Medicine, Baltimore, Maryland, United States of America; 2 Department of Ophthalmology, Johns Hopkins University School of Medicine, Baltimore, Maryland, United States of America; 3 Department of Cell Biology, Johns Hopkins University School of Medicine, Baltimore, Maryland, United States of America; 4 All India Institute of Medical Sciences, New Delhi, India; University of Reading, United Kingdom

## Abstract

Keratoconus (KC) is a complex thinning disease of the cornea that often requires transplantation. The underlying pathogenic molecular changes in this disease are poorly understood. Earlier studies reported oxidative stress, metabolic dysfunctions and accelerated death of stromal keratocytes in keratoconus (KC) patients. Utilizing mass spectrometry we found reduced stromal extracellular matrix (ECM) proteins in KC, suggesting ECM-regulatory changes that may be due to altered TGFβ signals. Here we investigated properties of stromal cells from donor (DN) and KC corneas grown as fibroblasts in serum containing DMEM: F12 or in serum-free medium containing insulin, transferrin, selenium (ITS). Phosphorylation of SMAD2/3 of the canonical TGFβ pathway, was high in serum-starved DN and KC fibroblast protein extracts, but pSMAD1/5/8 low at base line, was induced within 30 minutes of TGFβ1 stimulation, more so in KC than DN, suggesting a novel TGFβ1-SMAD1/5/8 axis in the cornea, that may be altered in KC. The serine/threonine kinases AKT, known to regulate proliferation, survival and biosynthetic activities of cells, were poorly activated in KC fibroblasts in high glucose media. Concordantly, alcohol dehydrogenase 1 (ADH1), an indicator of increased glucose uptake and metabolism, was reduced in KC compared to DN fibroblasts. By contrast, in low glucose (5.5 mM, normoglycemic) serum-free DMEM and ITS, cell survival and pAKT levels were comparable in KC and DN cells. Therefore, high glucose combined with serum-deprivation presents some cellular stress difficult to overcome by the KC stromal cells. Our study provides molecular insights into AKT and TGFβ signal changes in KC, and a mechanism for functional studies of stromal cells from KC corneas.

## Introduction

Keratoconus is a heterogeneous disease, with familial and environmental influences and multiple genes are suspected to have small effects in its pathogenesis [1]. Patients show thinning and steepening of the cornea, irregular astigmatism, decreased visual acuity, and corneal protrusion [2]–[7]. It affects both genders, usually with onset at puberty and progression through the mid-forties [8]–[11]. While genetic contributions in keratoconus are evident, suggestive *loci* and genes have yet to be validated and confirmed [12]. Pathogenic underpinnings include oxidative stress, connective tissue dysfunction, inflammatory changes, extracellular matrix (ECM) degradation, and association with contact lens [13]–[21]. Recently, we conducted a mass spectrometric proteomic analysis of the cornea and found decreased levels of several stromal ECM proteins including fibrillar collagens and proteoglycans [22]. Keratocytes, the resident stromal cells, produce and maintain the stromal ECM, responsible for more than 70% of the refractive power of the eye [23]. Studies are beginning to focus on these cells to gain deeper insights into the stromal degeneration seen in keratoconus.

Keratocytes are specialized neural crest-derived mesenchymal cells [24]–[27]. Isolated *in vitro,* keratocytes retain their typical dendritic phenotype under serum-free or serum-poor conditions and produce ECM proteoglycans and collagens typically seen in the native cornea [26], [28]. The keratocytes differentiate to fibroblasts after serum exposure [28], and to myofibroblasts in the presence of excess exogenous transforming growth factor beta 1 (TGFβ1) [29], with each cellular phenotype having distinctive gene expression patterns [30], and biomarkers in culture [31], [32].

The keratoconic stroma is associated with haze, reduced ECM proteins, fewer keratocytes and abnormal cellular morphology, all indicative of pathogenic changes in keratocytes [3], [33], [34]. The cellular pathophysiology is poorly understood at the molecular level. Here we show that stromal cells from keratoconus corneas expanded as fibroblasts, and serum-starved, have a dendritic morphology seen in primary keratocytes. The serum starved DN and KC fibroblasts show similar growth patterns. However, the KC cells display altered AKT and TGFβ signals that may relate to pathogenic changes in metabolic properties and decreased ECM as seen in the KC cornea. In addition, primary KC stromal cells, without prior expansion as fibroblasts, showed poor survival in serum-free media.

## Experimental Procedures

### Ethics Statement

KC corneas were obtained from patients undergoing keratoplasty at the Wilmer Eye Institute Cornea Service. Patients recruited for this study provided written informed consent for the use of their corneal tissues under a Johns Hopkins Medicine IRB approved protocol entitled “Genotypic and Phenotypic Assessment of Keratoconus (NA-00006544). Normal donor anterior stromal caps in Optisol –GS (Bausch & Lomb, Rochester, NY) were obtained from endothelial keratoplasty from Tissue Banks International (Baltimore, MD) and the Indiana Lions Eye and Tissue Bank (Indianapolis, IN) under established guidelines related to informed consent for research use of human donor corneas.

### Stromal Cell Isolation

Primary stromal cells were isolated as described before [32], [35]. Central corneal buttons were rinsed in cold Hanks balanced salt solution (HBSS; CellGro, Manassas, VA) supplemented with antibiotics (100 IU/ml Penicillin and 100 μg/ml Streptomycin), and incubated in 1mg/ml collagenase type L (C8176; Sigma Aldrich, St. Louis, MO) for 1 hour, the epithelium and endothelium were scraped off, the stroma cut into small pieces and digested in fresh 1 mg/ml collagenase-L for 1–3 hour at 37°C or overnight at 4°C for donor corneas. The digests were centrifuged at 500×*g* for 5 minutes and the pellet resuspended in a 10 mg/ml collagenase-L solution for a final digestion at 37°C with shaking at 150 rpm for 2 hours. All digested corneas were filtered through a 70 µm cell strainer (BD Falcon, Bedford, MA) and the isolated keratocytes centrifuged at 500×*g* for 5 minutes. The cell pellet was washed and plated in DMEM: F12 with ITS, 10 ng/ml FGF2, 1 mM *L*-ascorbic acid 2-phosphate sesquimagnesium salt hydrate (Sigma-Aldrich, St. Louis, MO) and 1% Penicillin/Streptomycin.

Alternatively, to directly establish fibroblasts, the stroma pieces were digested in 2 mg/ml of collagenase type-I (Invitrogen; Carlsbad, CA) in DMEM: F12 (Invitrogen,) supplemented with 5% fetal bovine serum (FBS), 1% antibiotics, 1 mM *L*-ascorbic acid 2-phosphate sesquimagnesium salt hydrate at 37°C with shaking until digestion was complete (∼3 hours). The suspension was then pelleted at 500×*g* for 5 minutes. Cells were then dissociated by re-suspending the pellet in 0.25% Trypsin-EDTA (Invitrogen) solution for 5 minutes and passed through a 40 µm cell strainer (Fisher; Waltham, MA). Finally, the trypsin solution was neutralized with serum containing DMEM: F12, and cells pelleted at 500×*g* for 5 minutes before being resuspended in the same medium and seeded into a T75 tissue culture flask (Nunc; Thermo Scientific, Waltham, MA) and returned to the tissue culture incubator. The Media was replaced every two days until 70% confluence was reached. The fibroblasts were expanded for a further passage and cryopreserved in 10% DMSO in DMEM: F12 containing 20% FBS.

### Cell Proliferation

Four individual patient and four patient (KC) fibroblasts were expanded in DMEM: F12, 5%FBS and 1% antibiotics and trypsinized, 10^4^ cells were seeded in triplicate wells of 24 well plates and allowed to adhere overnight. The fibroblasts were then washed twice in PBS and switched to the following serum-free media, all containing 1× ITS and 1 mM phosphoascorbic acid, 1) DMEM: F12, 2) high glucose serum-free DMEM (HGSF), and 3) low glucose serum-free DMEM (LGSF) for 4 days with a media change at day −2. These serum-starved fibroblasts were then washed and maintained in their respective media containing either 1× ITS, or 2 ng/ml TGFβ1 or both for up to 7 days. To assess cell number a stock solution of 0.5 mM Alamar blue (Sigma-Aldrich, R7017–5G) in PBS was diluted 1∶10 in culture media and incubated for 3 hours in the tissue culture incubator, fluorescence at 590 nm (excitation 530 nm) was measured on an EnVision 2104 multi-label reader (Perkin Elmer, Waltham, MA). Cell number was derived from standard curves generated separately with donor and patient samples with a dynamic range of 5×10^3^–2×10^5^ cells.

### RNA isolation and quantitative real time reverse transcription polymerase chain reaction (qRTPCR)

Cells were harvested, centrifuged and resuspended in 500 ul Trizol (Invitrogen). Total RNA was isolated using the RNAeasy Mini Kit (Qiagen, Valencia, CA) according to the manufacture's protocol. Quantitative RT-PCR was performed using SYBR Green PCR Master Mix and analyzed on a QuantStudio 12 K Flex System (Life Technologies). Target genes were amplified in 25 µl reaction volumes for 1 min at 95°C followed by 40 cycles of 15 seconds at 95°C, 30 seconds at 60°C and 1 min at 72°C and a final extension for 5 min at 72°C. Reactions were analyzed in triplicate, using the Expression Suite Software (Life Technologies) and mRNA fold changes calculated after normalizing to *GAPDH*. All primers were designed using the Primer 3 software and obtained from Integrated DNA Technologies (Coralville, IA). The following primers were obtained for *ALK1* (192 bp product) forward: 5′ –CTGGCTCTGAGGCTAGCTGT and reverse 5′ –GTAATCGCTGCCCTGTGAGT, for *ALK5* (104 bp product) – Forward: 5′ –GAGCATGGATCCCTTTTTGA and reverse 5′ –ATGTGAAGATGGGCAAGACC, and *GAPDH* (185 bp product) –5′ –GAGTCAACGGATTTGGTCGT and reverse 5′ –GACAAGCTTCCCGTTCTCAG.

### Western Blot Analysis

Cells were lysed in RIPA buffer (50 mM Tris-HCl, 150 mM NaCl, 2 mM EDTA, 1% NP-40 0.5% deoxycholate, 0.1% sodium dodecyl sulfate) and protein concentration measured using the BCA Assay System (Thermo Scientific). Samples were loaded in 10% polyacrylamide gels, 20 µg/lane and analyzed by SDS-PAGE followed by Immunoblotting. The following primary antibodies were used: anti-pSMAD2/3 and anti-pSMAD1/5/8 (Cell Signaling Technology, Beverly, MA), anti-p21 (monoclonal IgG, BD Biosciences), and pAKT1-ser473 (#4060, Cell Signaling Technology) and anti-GAPDH (Santa Cruz, Dallas, TX). Bands of interest were quantified using Image J 1.47 (National Institute of Health, Bethesda, USA).

### Microscopy

Images were taken in a Zeiss AXIO Observer A1 Microscope (Carl Zeiss) with an Olympus DP72 Camera (Olympus Imaging America, USA).

### Statistical methods

Each experiment was repeated two to four times as indicated in the figure legend. Statistical significance was assessed using unpaired Student's t test (Graphpad Prism) and p≤0.05 taken to be significant for two group experiments, one-way ANOVA with Tukey's multiple comparison tests for multiple test groups and two-way ANOVA for grouped datasets.

## Results

### Altered TGFβ signals in KC keratocytes

**Figure 1:**
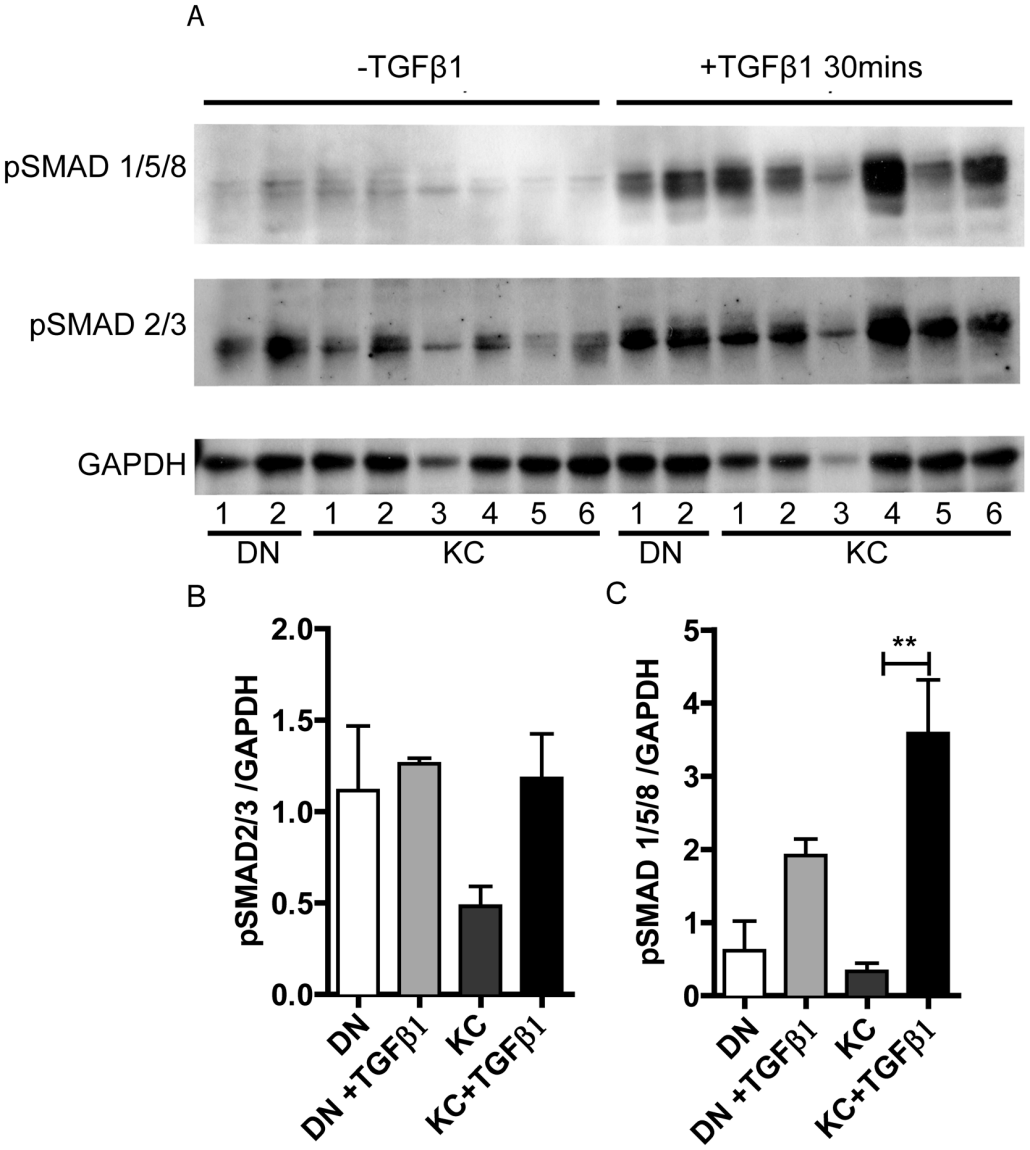
TGFβ signals in DN and KC keratocytes. Two DN and 6 KC keratocyte cultures were treated with TGF β1 (2 ng/ml for 30 minutes) and were immunoblotted for pSMAD2/3, pSMAD1/5/8 and GAPDH (A). Densitometry scans of relevant bands were normalized to GAPDH and compared by one-way ANOVA and * indicates p≤0.05 (B and C).

Our previous proteomic analyses of the corneal stroma had shown decreases in several TGFβ regulated ECM proteins in keratoconus (KC) corneas suggesting diminished TGFβ signals [22]. Therefore, we investigated TGFβ signals in stromal cells from control donor (DN) and KC corneas. The canonical TGFβ pathway involves the phosphorylation of SMAD2/3 after exposure to TGFβ ligands, binding of pSMAD2/3 with co-SMAD4 and transport of the complex to the nucleus for subsequent activation of specific subsets of genes [36]. We investigated the status of TGFβ signal transduction by assessing phosphorylated SMAD2/3 and SMAD1/5/8 after TGFβ1 treatment of serum-starved fibroblasts (Fig. 1). Stromal cells from individual KC and DN corneas were cultured in serum-free DMEM: F12 with ITS and phosphoascorbic acid, and ITS removed before treating with TGFβ1. We found that SMAD2/3 was already phosphorylated at high levels before the addition of exogenous TGF β1, with only a small increase after TGF β1 treatment (Fig. 1 A, C). On the other hand, SMAD1/5/8 was minimally phosphorylated under basal conditions in serum-starved DN or KC fibroblasts (Fig. 1 A), and markedly phosphorylated after 30 minutes (Fig. 1 A, B). The increase in pSMAD1/5/8 over its basal level in KC was significantly higher than DN cells. Usually, SMAD1/5/8 phosphorylation occurs after BMP ligand engagement with their Type I and subsequently Type II receptors. However, in endothelial cells an unconventional signaling by TGFβ1 involves the phosphorylation of SMAD 1/5/8 [37]. The TGFβ1-pSMAD1/5/8 is therefore a novel axis in corneal keratocytes.

**Figure 2:**
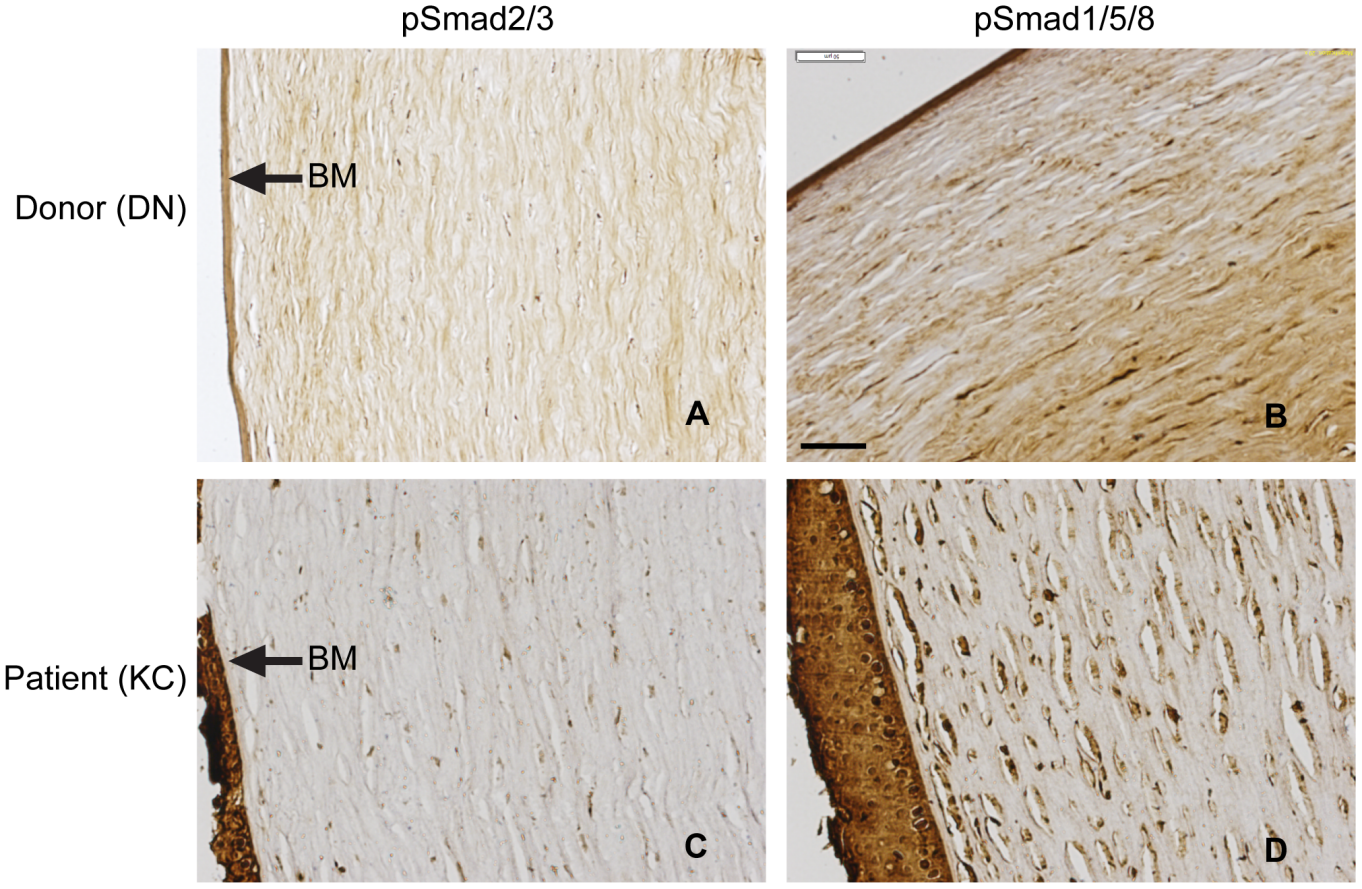
Immunohistochemistry of pSMAD2/3 and pSMAD1/5/8 in DN and KC corneas. DN stromas were obtained from stromal caps processed for endothelial keratoplasty and thus missing the epithelial (EPI) layers as these are removed during this procedure, but retain the Bowman's membrane (BM). Scale bar  = 100 µm. The results shown are representative of two donor and three patient corneas.

We tested for the presence of pSMAD2/3 and pSMAD 1/5/8 in sections of DN and KC corneas by immunohistochemistry (IHC) (Fig. 2). In general, the IHC staining of pSMAD1/5/8 was stronger than that of pSMAD2/3, with pSMAD2/3 staining the stroma diffusely, while pSMAD1/5/8 stained the keratocyte cell bodies.

**Figure 3:**
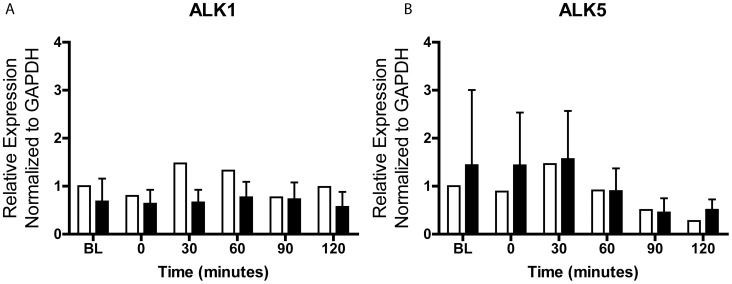
*ALK1* and *ALK5* expression in DN and KC reverted-keratocytes. Serum-starved fibroblasts maintained in DMEM: F12/ITS/phosphoascorbic acid for 4 days were switched to DMEM: F12 without ITS (BL), 2 ng/ml TGF β1 was added and cells harvested at 0–120 min for total RNA isolation. Fold change in gene expression relative to *GAPDH* was calculated as 2^−ΔΔCt^.

To further understand the unusual constitutively high pSMAD2/3 and inducible pSMAD1/5/8 signaling axes we investigated the expression of TGFβ/BMP pathway-defining receptor transcripts in serum-starved fibroblast from DN and KC samples. There are 5 Type II and 7 Type I receptors that regulate TGFβ superfamily ligand recognition [36], [38]. TGFβ binds to the Type I TGFβ R1 also known as ALK5 (Activin receptor-like kinase), and the Type II TGFβ R2, while Activin, Nodal and BMPs involve the type II receptors ACVR2A and ACVR2B. The ACVRL1 or ALK1 is the type I receptor normally used by BMP9 and 10; but its induction by TGFβ has been noted in endothelial cells in high TGFβ ligand concentrations [39], [40]. The TGFβ – pSMAD2/3 axis requires ALK5, whereas the less common TGFβ -pSMAD1/5/8 signaling arm requires ALK1. By qRTPCR we found that both ALK1 and ALK5 were constitutively expressed, with no significant differences in their expression between serum-starved DN and KC fibroblasts (Fig. 3).

**Figure 4:**
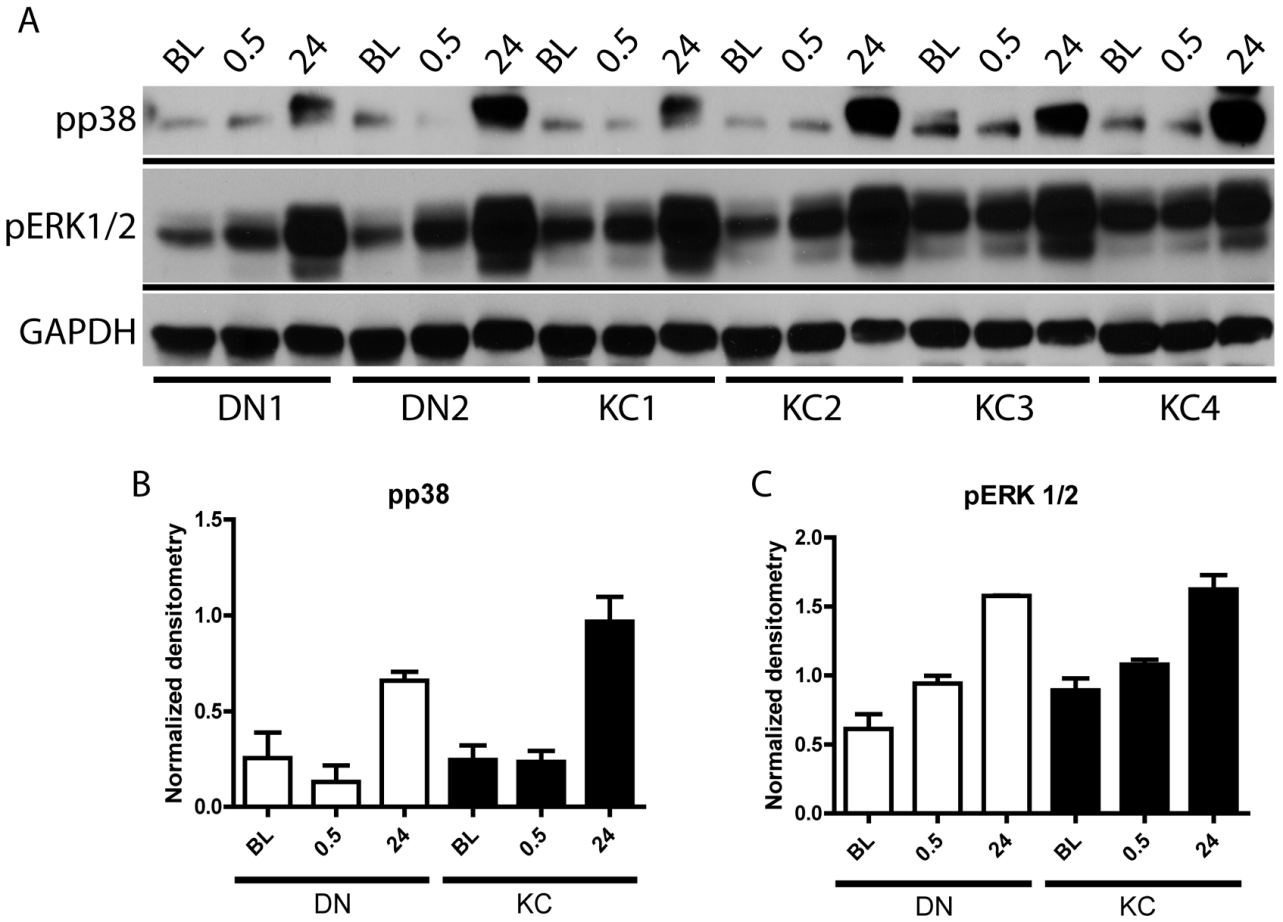
Non-canonical TGF β signals were activated by 24 hours of TGF β treatment. Serum-starved fibroblasts were treated with 5 ng/ml of TGFβ1, unstimulated (BL) and treated cell were harvested after 0.5 and 24 hours. (A) Total protein extracts were immunoblotted for phosphorylated ERK1/2 (pERK 1/2), phosphorylated p38 (pp38) MAPK and GAPDH. (B–C) Densitometry quantification of pp38 and pERK 1/2.

We explored TGFβ non-canonical signaling events by treating serum-starved fibroblasts with TGFβ1 (5 ng/ml) and immunoblotting the cell extracts for phosphorylated ERK1/2 (extracellular signal regulated kinase) and p38 MAPK (mitogen activated protein kinase). After 30 minutes of TGFβ1-stimulation, the phosphorylated form of p38 was minimally present; by 24 hours it was very high in DN and KC serum-starved fibroblasts, indicating that the non-canonical pathways were equally active in DN and KC cells (Fig. 4).

### Growth and proliferation of donor and KC keratocytes

**Figure 5:**
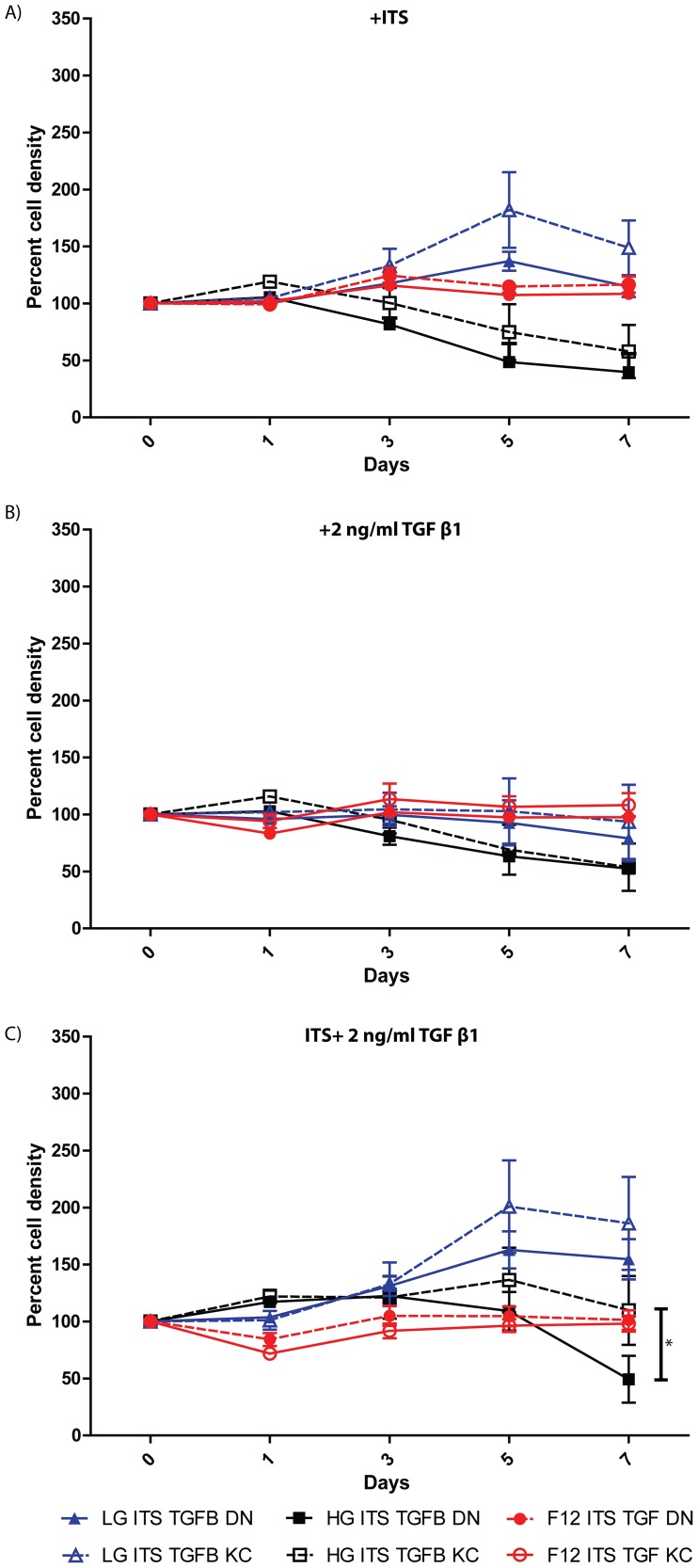
Differential growth response of serum-starved DN and KC fibroblasts to ITS and TGF β1 in hyperglycemic and normoglycemic media. Four individual DN and KC reverted keratocytes were placed in serum-free LGSF, HGSF or DMEM: F12 supplemented with (A) ITS, (B) 2 ng/ml TGFβ1 or (C) both together and proliferation assessed over 7 days. The results shown are the mean ± SEM of 4 independent DN and KC serum starved corneal fibroblasts, with 3 technical replicates of each, at each time point. Significance was calculated using two-way ANOVA and multiple comparisons with * indicating p≤0.05.

A number of earlier studies using different approaches came to the conclusion that keratocyte density and morphology were altered in KC and that they displayed increased cell death [22], [41]–[45]. Here we tested the growth and proliferation (Fig. 5) of 4 individual DN and 4 KC samples in serum free media Low-glucose (LGSF), high-glucose (HGSF) or DMEM: F12 (F12) serum free media containing insulin, transferrin and selenium (ITS) and /or TGF β1, by measuring viable cell content over 7 days. In general serum-starved DN and KC fibroblasts behaved in a similar fashion – both showed increased growth in ITS-containing LGSF (Fig. 5A). A previous study identified LGSF (5.5 mM glucose) DMEM as most suitable for the conversion of human corneal fibroblasts to keratocytes [46]. The ITS supported growth advantage seen in LGSF was not lost altogether in intermediate glucose containing DMEM: F12; whereas in HGSF cell loss was evident (Fig. 5A). TGFβ1 suppressed growth and there was very little increase in cell content in either LGSF (low glucose) or DMEM: F12 (intermediate glucose), while viable cell content decreased in HGSF. In the presence of ITS however, the growth suppressive effects of TGFβ1 were abrogated in low glucose media (Fig. 5B). Both DN and KC serum-starved cells survived poorly in HGSF, while DMEM: F12 appeared to buffer cells from growth promoting or suppressing effects of ITS and TGFβ1, respectively. With respect to cell morphology, DN (Figure S1) and KC (not shown) stromal cells appeared similar, with cells showing a tendency to pile up in DMEM: F12/ITS. Serum-starved DN and KC fibroblasts imaged after three weeks in LGSF and ITS showed extensive networks of ECM, with the KC cells appearing somewhat larger (Figure S2).

**Figure 6:**
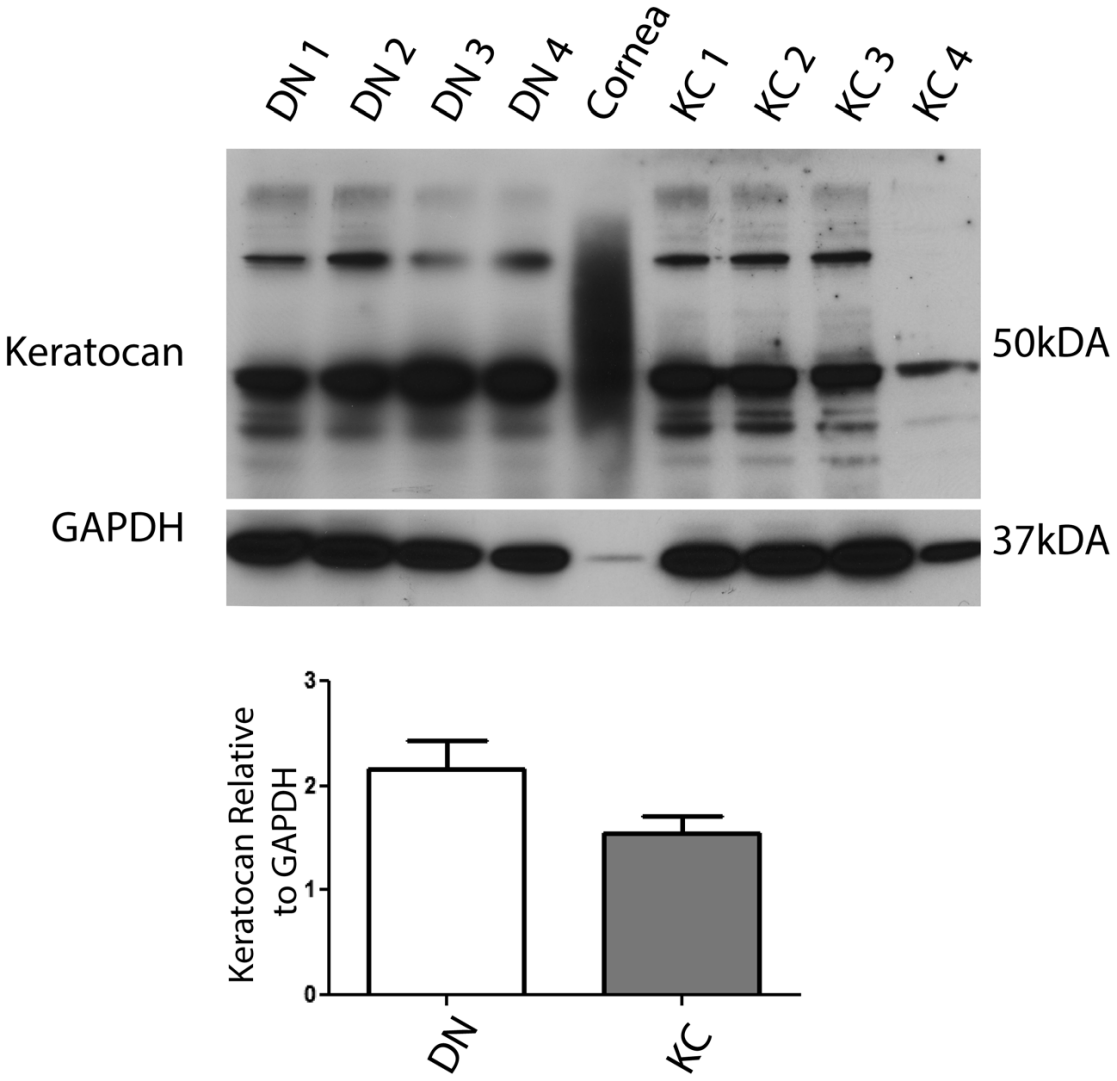
Keratocan was detected in DN and KC serum-starved fibroblasts. Four independent DN and KC serum starved corneal fibroblasts grown in LGSF for 2 weeks were extracted and immunoblotted for keratocan. The cells produce a discreet band of minimally glycosaminoglycanated keratocan core protein while the corneal tissue extract shows a broad keratocan proteoglycan band.

The keratocan core protein is an accepted marker for the keratocyte phenotype [47]. Since LGSF DMEM and ITS yielded long term growth, we tested for the presence of keratocan to assess the extent of phenotype retention under these culture conditions (Fig. 6). Keratocan levels were equally strong in all DN and KC serum-starved corneal fibroblasts, indicating their similarities to keratocytes. An extract of whole cornea, used as a positive control showed a smeared keratocan band typical of the heterogeneous glycosaminoglycanated proteoglycan forms of the core protein. In cell culture these proteoglycans are often not fully modified with glycosaminoglcans as they are in the cornea, and hence the sharp nature of the band in the cell culture extracts.

**Figure 7:**
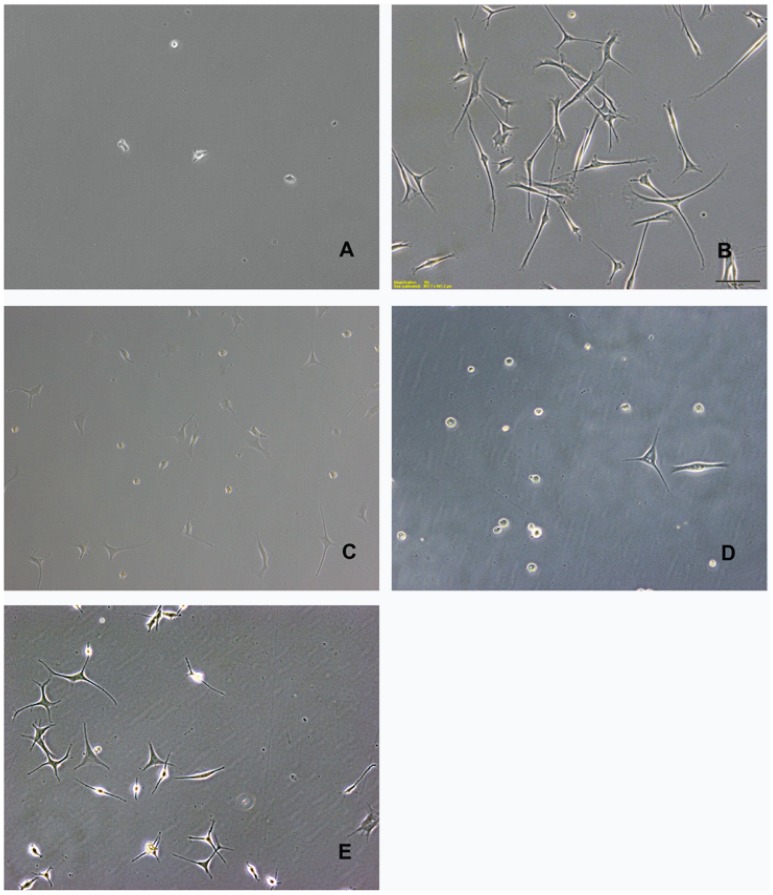
Primary keratocytes, from KC corneas show poor survival in serum-free medium. Stromal cells from DN (A and B) and KC (C–E) corneal buttons plated in serum-free DMEM: F12 and maintained in the same with ITS, FGF2 and phosphoascorbic acid for 2 days (A and C) were equally attached and had similar morphologies. Primary DN keratocytes at 2 weeks after plating had a typical dendritic morphology (B), while KC keratocytes showed many rounded detaching cells by 12 days (D). At an earlier time point (6 days) the KC keratocytes showed a healthy dendritic morphology. Results are representative of four KC and 2 DN primary cell isolates.

In our earlier attempts to generate primary keratocytes from KC corneas, collagenase-extracted cells were directly plated in growth factor- and serum-free DMEM: F12 medium without their prior expansion as fibroblasts, and subsequently placed in DMEM: F12 with ITS, FGF2 and phosphoascorbic acid. These conditions were previously described as most appropriate for keratocyte growth [48]. By 48 hours the plated cells had attached and begun to show a dendritic morphology (Fig. 7A and C). By 2 weeks, the primary DN cells (not passaged) continued to grow in serum-free DMEM: F12/ITS and maintain their keratocyte morphology (Fig. 7B), while KC keratocytes that were morphologically similar to DN keratocytes early on, at 6 days after isolation (Fig. 7E), became rounded and began to deteriorate by 12 days (Fig. 7D). Thus, freshly isolated KC cells showed poor survival in serum-free medium, mimicking metabolic and growth impairment features of *in situ* keratocytes from KC corneas.

### AKT signal transduction changes in KC stromal cells

**Figure 8:**
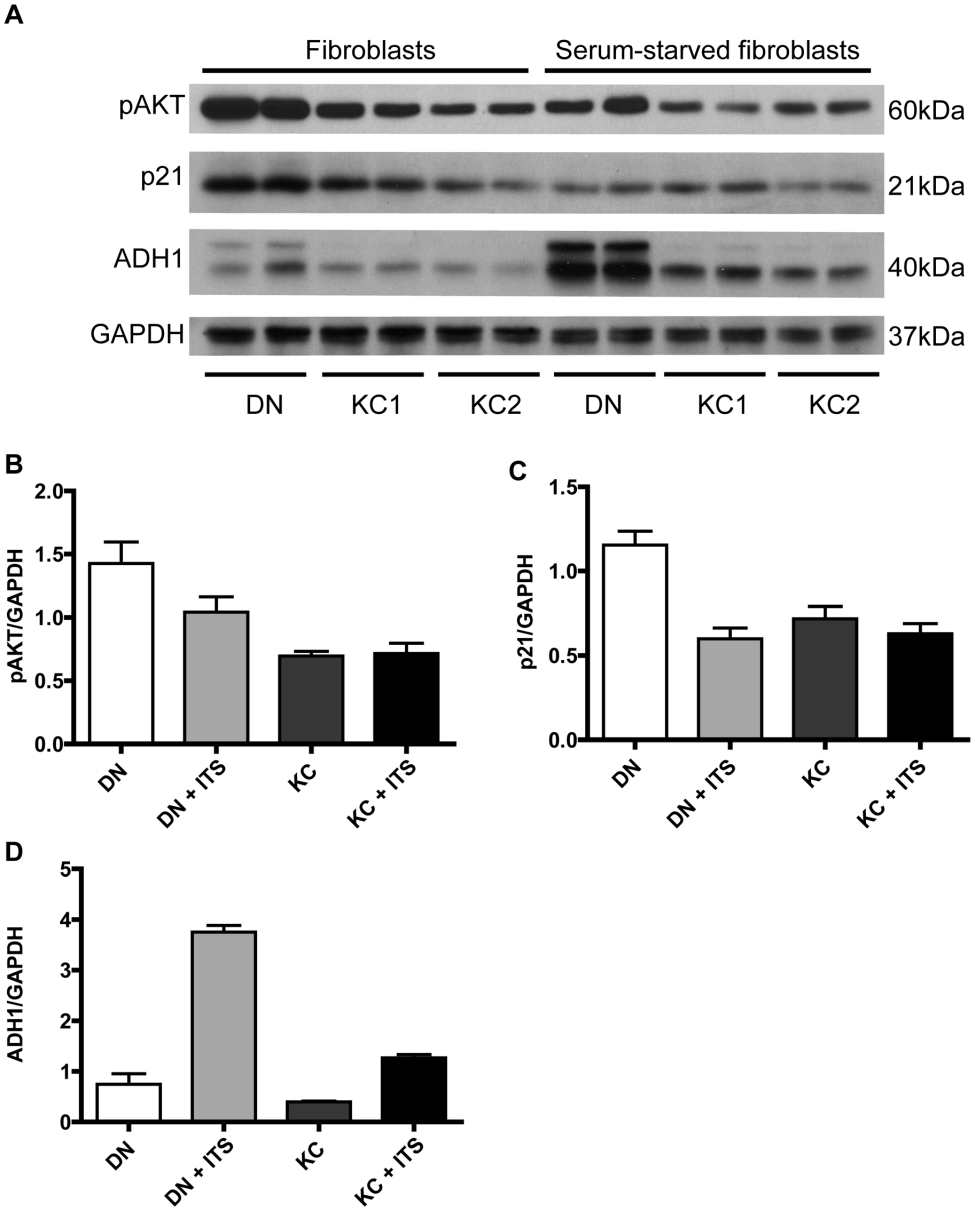
Reduced pAKT and metabolic activities in KC fibroblasts in DMEM: F12. DN and KC stromal fibroblasts in the presence or absence of serum in DMEM: F12+ ITS were extracted and immunoblotted for pAKT (phosphorylated at serine-473), p21, alcohol dehydrogenase 1 (ADH1) and GAPDH (A). Band intensities (B–D) were measured using Image J, normalized to GAPDH and shown as histograms.

**Figure 9:**
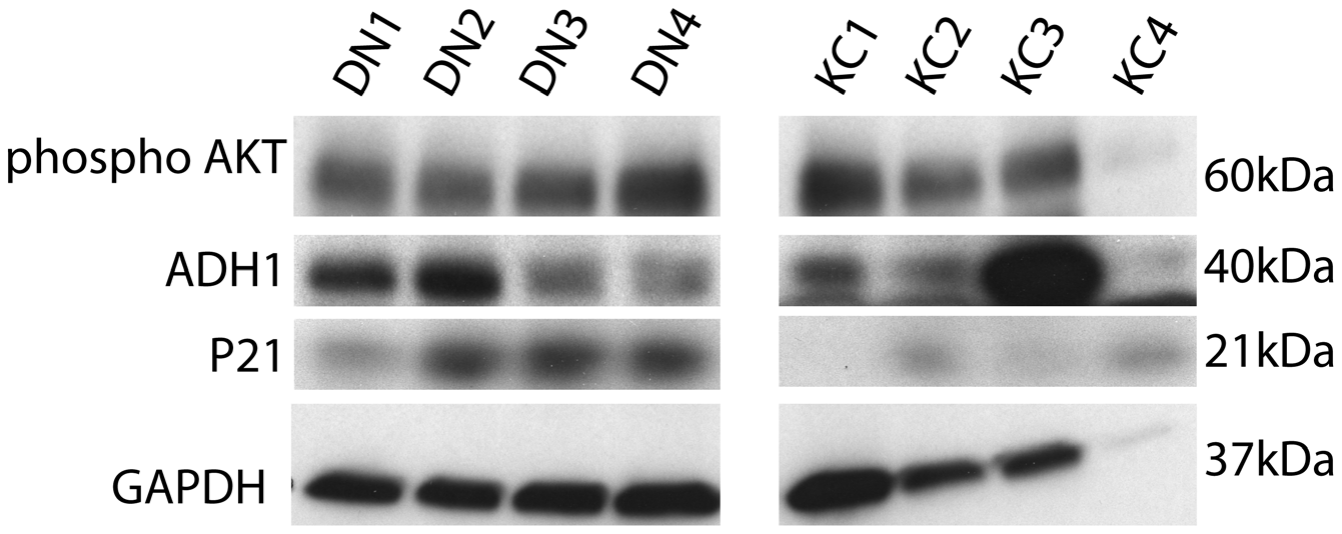
Similar pAKT in DN and KC fibroblasts in LGSF/ITS growth medium. Four individual DN and KC serum starved corneal fibroblasts were grown in LGSF DMEM with 1× ITS, 1 mM phosphoascorbic acid and 1% antibiotic for 2 weeks. The cell extracts were analyzed for pAKT, ADH1, p21 and GAPDH by SDSPAGE and immunoblotting.

We considered whether AKT signal alterations contribute to keratocyte growth and survival deficiencies in the pathogenesis of keratoconus. Our proteomic studies had also identified increases in ER stress and apoptotic proteins, and decreases in multiple collagens and proteoglycans [22]. Three closely related serine/threonine kinases, AKT1–3, form a key node downstream of growth factor and receptor tyrosine kinases and cytokines and regulate proliferation, cell survival, growth and metabolism [49]. AKT activation ensues with phosphorylation of the threonine-308 and subsequent phosphorylation of serine-472/473/474 by mTOR1. In both DN and KC fibroblasts (in serum-containing DMEM: F12), activated pAKT was elevated (Fig. 8); this was expected as serum contains growth factors, including platelet-derived growth factor, a strong activator of AKT [50]. Compared to DN, the KC fibroblasts showed lower levels of activated pAKT. In serum-starved fibroblasts in DMEM: F12/ ITS, pAKT was uniformly low in DN and KC samples. Since long-term survival and keratocyte-like phenotype retention and keratocan marker synthesis was better in LGSF/ITS medium, we examined pAKT levels in this growth condition as well and found no consistent difference between DN and KC samples (Fig. 9). Class I alcohol dehydrogenase (ADH1), an indicator of metabolic activity [51] was low in KC keratocytes in DMEM: F12, compared to DN keratocytes (Fig. 8). However, in LGSF DMEM: ITS there were no differences between DN and KC serum starved corneal fibroblasts, except one patient sample that had very high levels of ADH1 (Fig. 9). We also examined p21, an inhibitor of cyclin-dependent kinases and cell proliferation. The level of p21 was higher in DN fibroblasts, while serum-starved fibroblasts showed no major difference (Fig. 8 and 9).

## Discussion

Here we developed methods to study stromal cells from individual DN and KC cornea button halves. We show that single cornea-half derived stromal cells could be expanded as fibroblasts and stored at low passage numbers. In serum-free media containing ITS and phosphoascorbic acid, the fibroblasts assumed a dendritic morphology typically seen in keratocytes.

We investigated TGF β signal transductions in serum-starved KC and DN fibroblasts, as multiple lines of evidence supported a role for this signaling network in keratoconus. The TGFβ1 and 2 ligands are present in their inactive form in the corneal epithelium under homeostatic conditions, while in their active cleaved forms they are present in the epithelium and to a lesser extent in the stroma during injury and infections [52]–[55]. Earlier studies have shown that keratocytes respond to TGF β1 stimulus, however these were conducted in the context of myofibroblastic changes, ECM production and fibrosis [56]–[59].

Serum-starved fibroblasts exposed to TGF β1 showed increased phosphorylation of SMAD1/5/8, and this phosphorylation was significantly higher in the KC cells. This less common route of TGFβ1 signal transduction reported before in vascular endothelial cells may be operative in the corneal stroma. Moreover, this appeared to be increased in keratoconus. The canonical signaling via phosphorylation of SMAD2/3 was constitutively high in DN and KC serum-starved fibroblasts with the KC cells showing higher levels that did not reach statistical significance. Constitutive high pSMAD2/3 could be due to the fact that these cells were expanded in serum containing medium where endogenous TGFβ levels could have been elevated. Presence of the transcripts for the receptor subunits ALK1 (TGFβ – pSMAD1/5/8 axis) and ALK5 (TGFβ – pSMAD 2/3 axis) in both serum-starved DN and KC fibroblasts indicates that the cells have the means to operate both signal axes. Immunohistochemistry of corneal sections also detected pSMAD2/3 and pSMAD1/5/8 in the stroma associated with keratocytes. The pSMAD1/5/8 staining appeared stronger in the KC corneas, although this remains to be tested in a larger set of DN and KC corneas. By IHC staining we had also detected increased staining of TGFβ2 and pSMAD2/3 in the epithelial layers of keratoconus corneas [60], but had not tested pSMAD1/5/8 in that study. It is not clear at the moment as to how increased phosphorylated intermediates may relate to overall TGFβ signal propagation. The TGFβ network is complex, and increased ligand or increased pSMAD signal intermediates do not necessarily correlate with increased signal propagation [38], [61]. Moreover, how these signaling axes regulate gene expression programs and contribute to reduced ECM proteins as seen in our proteomic study of KC [22] are not well understood. Interestingly, a group of heritable connective tissue diseases which include syndromic aortic aneurisms, including Marfan syndrome, Loeys-Dietz syndrome, Shprintzen-Goldberg syndrome, all associated with vessel wall weakening, have been traced back to genetic variants in multiple components of the TGFβ signal network [62], [63]. Keratoconus may share certain molecular pathways with these diseases, opening new avenues to research its pathogenesis.

A body of earlier work on bovine corneas have shown that primary stromal cells plated in serum-free DMEM: F12 with small amounts of platelet-poor horse serum or ITS displayed a dendritic morphology and synthesized keratocan, the corneal keratan sulfate proteoglycan considered to be a marker for keratocytes [26], [28], [48], [64]. Similarly, we recently showed that primary cells extracted from donor human corneas could be plated overnight in growth factor and serum-free DMEM: F12 for attachment and subsequently grown in the presence of ITS and phosphoascorbic acid expressed keratocan [32]. However, when we used this approach on freshly isolated stromal cells from keratoconus corneas, cell death was unacceptably high at the early stages and ultimately led to poor total cell yield. This is consistent with the idea that the keratocytes in keratoconus have some functional, metabolic deficiencies and a significant role in the thinning degenerative corneal phenotype of this disease [3], [18], [33], [34], [42]. Therefore, the growth potentials of patient and donor cells were further investigated using serum-starved fibroblasts that resemble keratocytes in their dendritic morphology and keratocan expression. We tested the effects of insulin (ITS) and TGFβ1 on cell proliferation in serum-starved fibroblasts in low (5.5 mM) and high (25 mM) glucose DMEM or DMEM: F12 with intermediate (17.5 mM) glucose to recreate normoglycemic and hyperglycemic conditions. We found that TGF β1 restricted or suppressed keratocyte growth in DN and KC keratocytes, and this growth suppression was counteracted by insulin in ITS. The negative regulation of cell cycle progression by TGF β is well documented in epithelial, hematopoietic and neural cells [36]. Studies on human corneal fibroblasts have also shown suppression by TGF β and promotion of growth by insulin [65]. Hyperglycemic conditions have been reported to induce oxidative stress in multiple cell types [66], [67]; combined with serum starvation it may be detrimental to serum-starved KC patient fibroblasts, in particular. Indeed, our cell proliferation assay showed loss of cells in high glucose DMEM, while low glucose medium seemed to alleviate this effect, and significantly so in patient serum-starved fibroblasts. Moreover, serum-starved fibroblasts from donor and patient corneas could be cultured long term in low glucose medium, where they synthesized keratocan and elaborated an ECM.

In summary, this study shows that stromal cells can be cultured and banked from individual donor and patient cornea halves and reverted to a keratocyte-like morphology for functional analyses. A novel TGFβ- pSMAD1/5/8 route was identified in the cornea. Corneal fibroblasts and serum-starved fibroblasts from keratoconus patients showed differential responses to insulin and TGF β1 that will be further investigated to elucidate disease-specific molecular changes.

## Supporting Information

Figure S1 **Reverted DN keratocytes in low and high glucose medium.** Reverted DN keratocytes have the typical dendritic morphology in ITS containing LGSF or DMEM: F12, but appear fibroblastic in HGSF/ITS and in all media containing TGFβ1. In DMEM: F12 containing both ITS and TGFβ1 the cells tend to pile up. Reverted KC keratocytes displayed similar properties under these culture conditions (not shown).
(TIF)

Figure S2 **Serum-starved DN and KC fibroblasts produce an ECM in long term cultures in LGSF/ITS.** After 3 weeks in culture maintained in LGSF/ITS, DN and KC cells produced a fine fibrous ECM (black arrows) with embedded cell bodies.
(TIF)
