# Correction: Transforming Growth Factor β and Insulin Signal Changes in Stromal Fibroblasts of Individual Keratoconus Patients

**DOI:** 10.1371/journal.pone.0126201

**Published:** 2015-04-17

**Authors:** The PLOS ONE Staff

There is an error in the legend for Fig 3. Please see the complete, corrected Fig 3 here.

**Fig 3:**
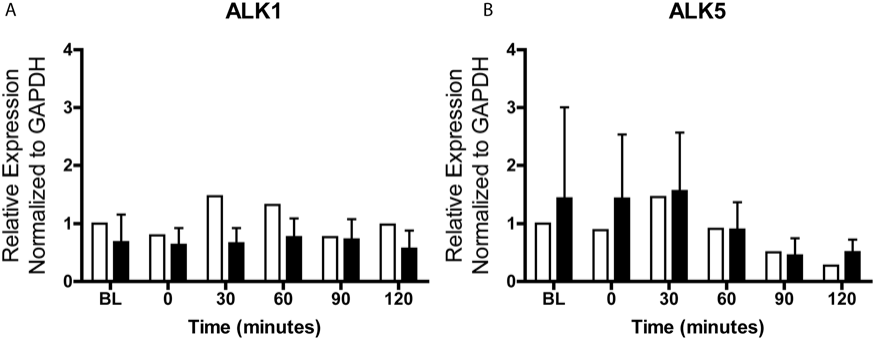
ALK1 and ALK5 expression in DN and KC reverted-keratocytes. Reverted donor (white bars) and patient (black bars) keratocytes maintained in DMEM:F12/ITS/phosphoascorbic acid for 4 days were switched to DMEM:F12 without ITS (BL), 2ng/ml TGFβ1 was added and cells harvested at 0–120 min for total RNA isolation. Fold change in gene expression relative to GAPDH was calculated as 2^-ΔΔCt^.

There is an error in the image for Fig 5. Please see the complete, corrected Fig 5 here.

**Fig 5:**
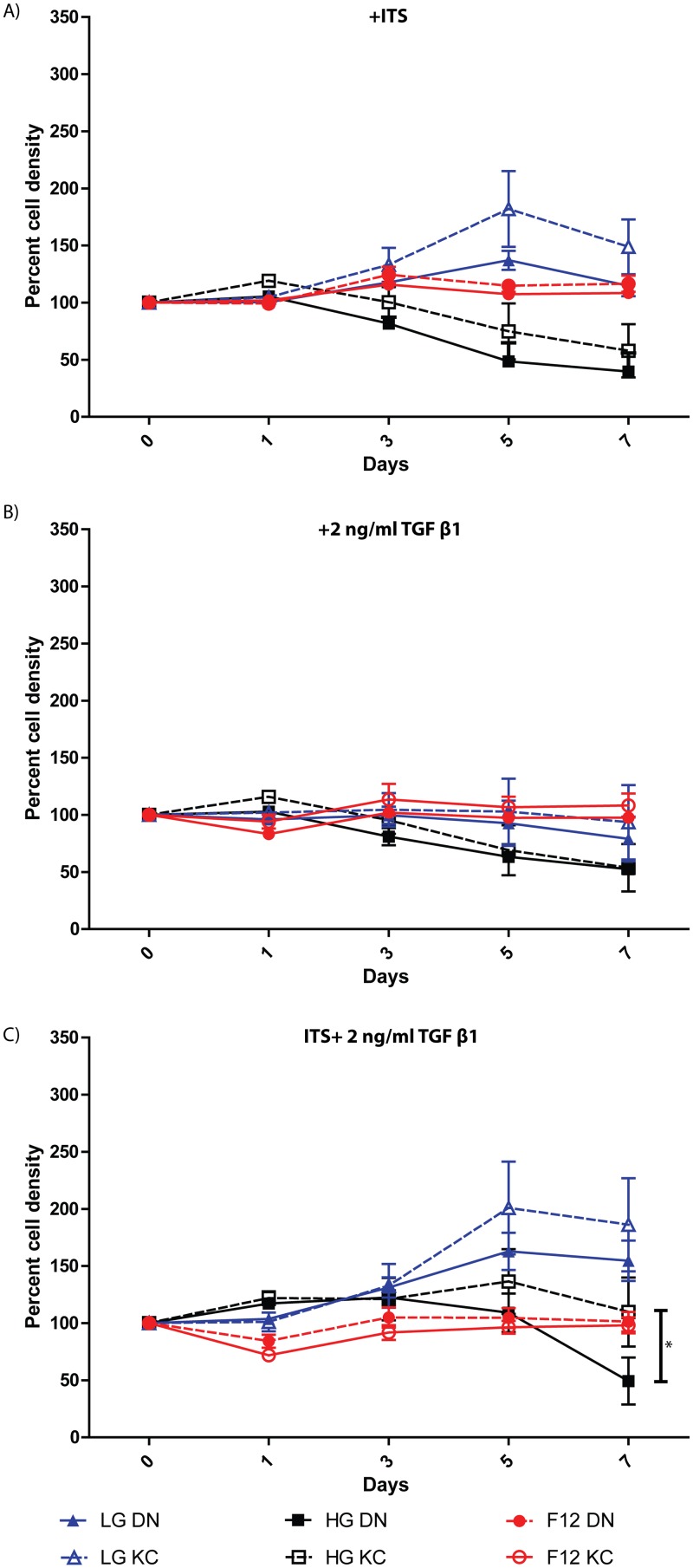
Differential growth response of reverted DN and KC keratocytes to ITS and TGFβ1 in hyperglycemic and normoglycemic media. Four individual DN and KC reverted keratocytes were placed in a) serum-free DMEM:F12, b) high glucose serum-free (HGSF) DMEM, and c) low glucose serum-free (LGSF) DMEM, each with ITS, TGFβ1 or both together and proliferation assessed over 7 days. The results shown are the mean ± SEM of 4 independent DN and KC primary stocks, with 3 technical replicates of each, at each time point. Significance was calculated using two-way Anova and multiple comparisons with * indicating p≤0.05.
