# Supplementary figures and images for: Transforming Growth Factor β and Insulin Signal Changes in Stromal Fibroblasts of Individual Keratoconus Patients
Source: PLoS One. 2014 Sep 23;9(9):e106556. doi: 10.1371/journal.pone.0106556 (PMC4172437; doi:10.1371/journal.pone.0106556)

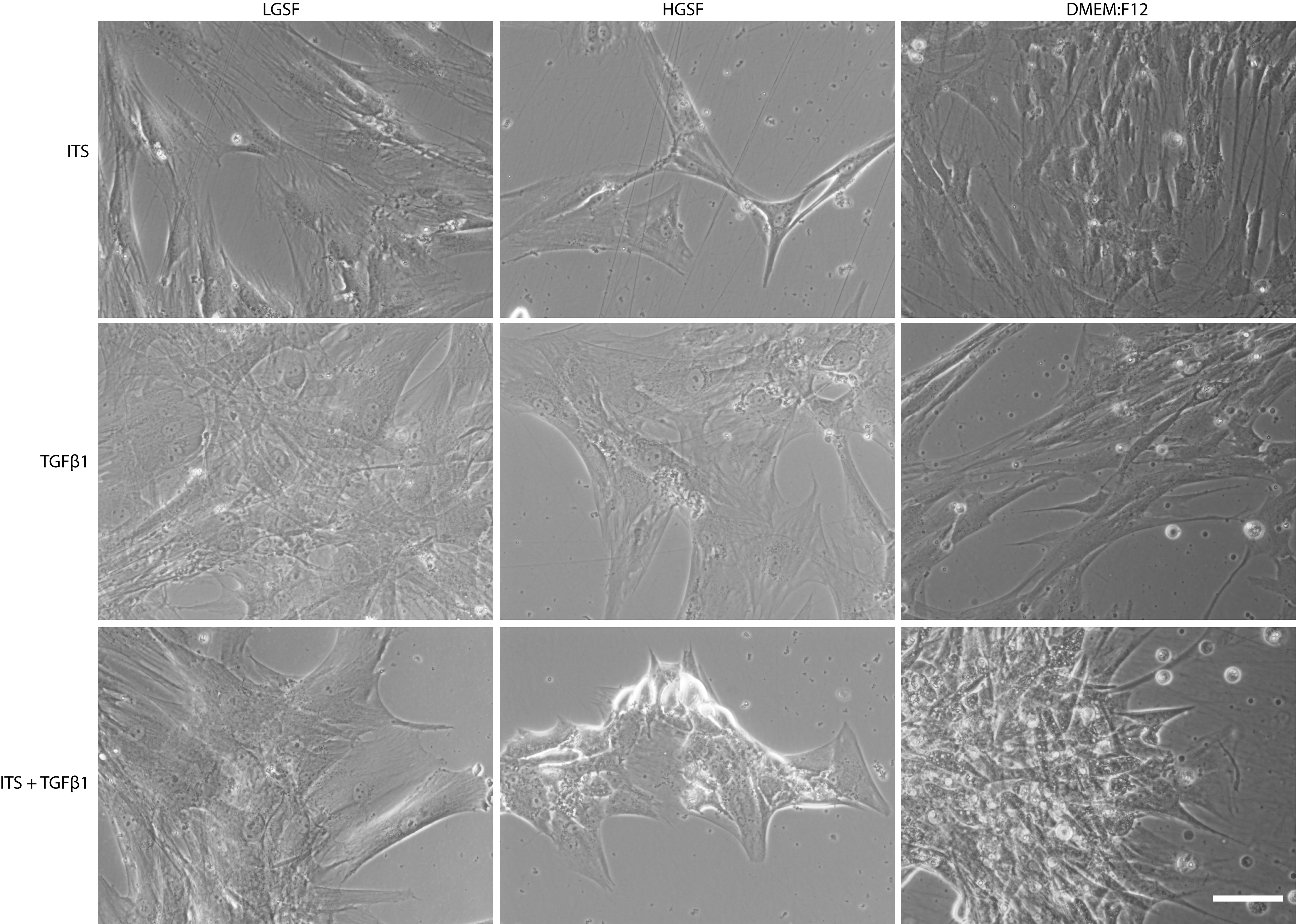

Supplement: Figure S1 — Reverted DN keratocytes in low and high glucose medium. Reverted DN keratocytes have the typical dendritic morphology in ITS containing LGSF or DMEM: F12, but appear fibroblastic in HGSF/ITS and in all media containing TGFβ1. In DMEM: F12 containing both ITS and TGFβ1 the cells tend to pile up. Reverted KC keratocytes displayed similar properties under these culture conditions (not shown). (TIF) [file pone.0106556.s001.tif]

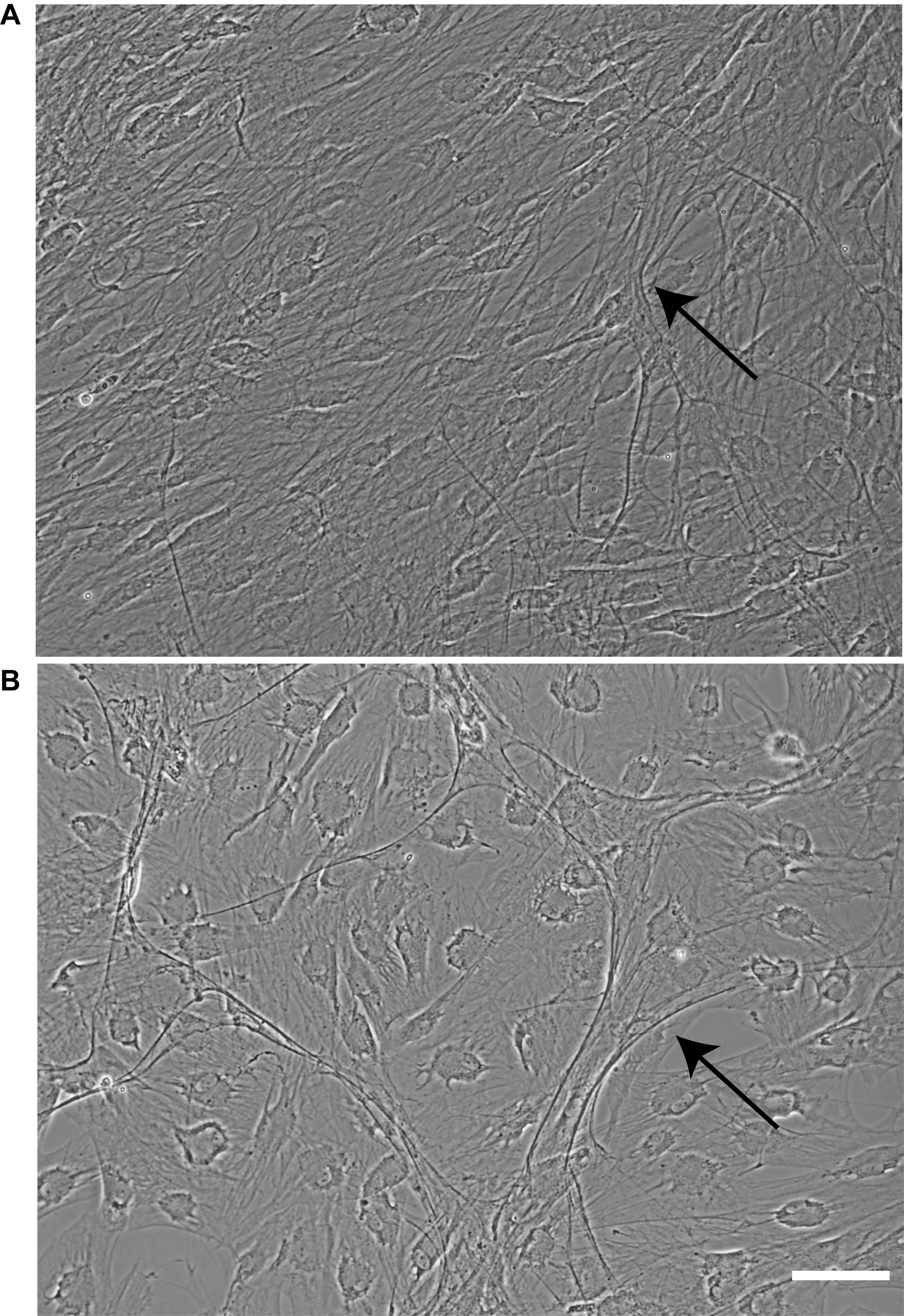

Supplement: Figure S2 — Serum-starved DN and KC fibroblasts produce an ECM in long term cultures in LGSF/ITS. After 3 weeks in culture maintained in LGSF/ITS, DN and KC cells produced a fine fibrous ECM (black arrows) with embedded cell bodies. (TIF) [file pone.0106556.s002.tif]
